# Long-term outcome of giant cell tumor of bone involving sacroiliac joint treated with selective arterial embolization and curettage: a case report and literature review

**DOI:** 10.1186/1477-7819-11-72

**Published:** 2013-03-18

**Authors:** XiuChun Yu, Ming Xu, SongFeng Xu, ZhiHou Fu

**Affiliations:** 1 Orthopedic Department, General Hospital of Jinan Military Commanding Region, Jinan, 250031, China

**Keywords:** Giant cell tumor of bone, Sacrum, Pelvis, Selective arterial embolization, Curettage, Long term outcome

## Abstract

**Background:**

Giant cell tumor of the sacrum, especially involving the sacroiliac joint, is rare, but is particularly challenging to treat. The long term outcome of a patient was studied with giant cell tumor involving the sacroiliac joint treated with selective arterial embolization and curretage.

**Method:**

One patient with giant cell tumor involving the sacroiliac joint was treated with selective arterial embolization and curettage in our hospital in October 2002. The curettage and bone grafting was done after two times of selective arterial embolization;1600 ml of blood were transfused and no complications developed during the operation.

**Results:**

At the final follow-up of 9 years after the operation, no local recurrence and metastasis developed and she retained normal activity in daily life.

**Conclusion:**

We think it is an optimal treatment for giant cell tumor involving the sacroiliac joint, with repeated selective arterial embolization and curettage, which has the advantage of less injury, less blood loss and fewer complications.

## Background

Giant cell tumor (GCT) is a locally aggressive, benign bone tumor with a high risk of local recurrence and a low risk of metastasis after treatment. Giant cell tumor of the sacrum, especially involving the sacroiliac joint, is rare, but is particularly challenging to treat since the tumor is frequently diagnosed late, and is often quite extensive within the bone and surrounds the sacral nerve roots. The sacral canal can accommodate large, slowly growing GCTs that become symptomatic only when they become large enough to compress adjacent nerves or pelvic organs. Patients often present with nonspecific low back pain. Treatment of GCT involving the sacroiliac joint is not straightforward. Excision of the affected sacral and iliac bone almost always results in loss of function of the sacral nerve roots with incontinence and lumbopelvic discontinuity. Resection of a considerable portion of the sacrum has a high incidence of neurological complications, which may affect bowel and bladder control and may lead to impotence in men [1]. Curettage alone is challenging due to blood loss and potential damage to nerve roots, and complete removal of the tumor is unlikely, with a high risk of recurrence [2]. Repeated selective arterial embolization (RSAE) of the tumor has had some success, but is usually used as a precursor to surgery to decrease bleeding [1,3,4].

In this manuscript, we report the long-term clinical outcome of a case with GCT involving the sacroiliac joint that was successfully managed by twice performing RSAE and curettage, and bone grafting. We stress the effectiveness of the procedure as being a less invasive and less complicated primary treatment for GCT of the sacrum and ilium. Informed consent was given before the operation and the patient was informed that data concerning the case would be submitted for publication.

## Case presentation

**Figure 1:**
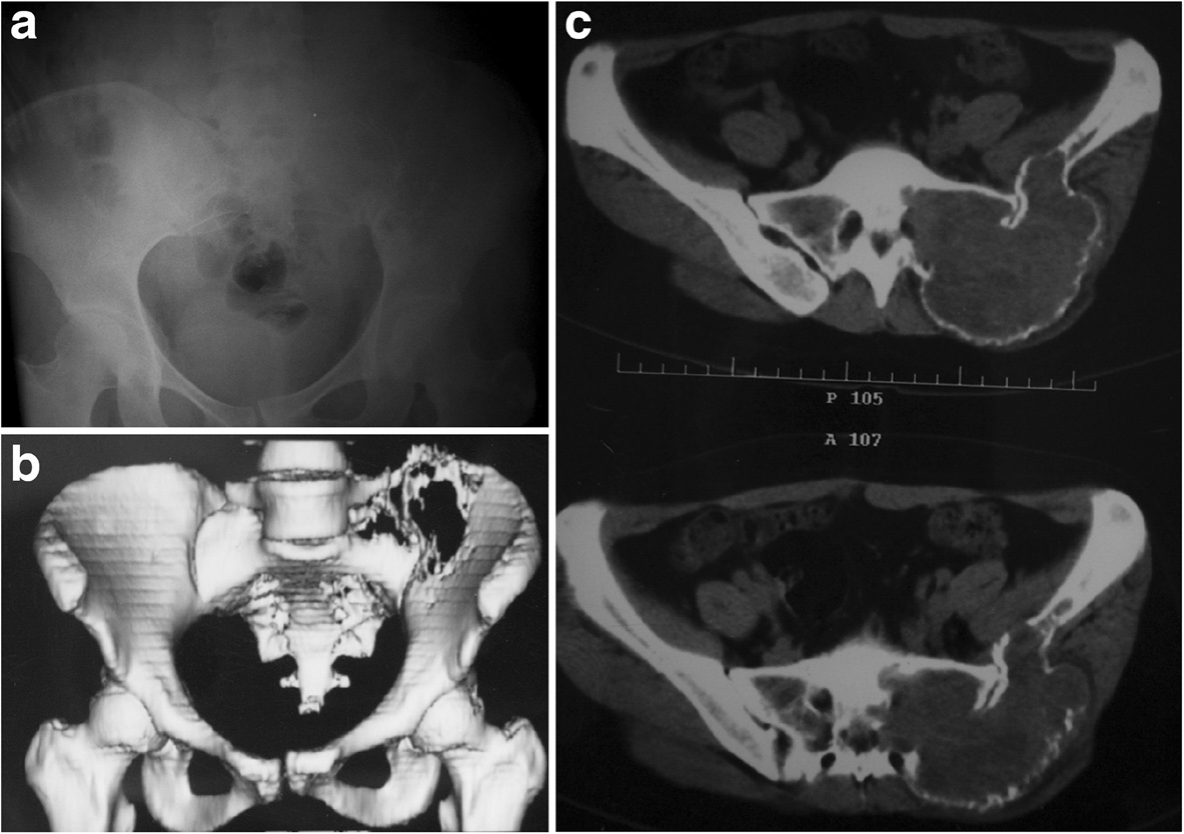
**A 31-year-old woman presented with giant cell tumor involving left sacroiliac joint.** Preoperative radiography (**a**) and computerized tomography (CT) (**b**) shows an eccentric, geographic, destructive, osteolytic lesion involving the sacrum and posterior superior iliac spine, with slight displacement of the pubic symphysis and left sacroiliac joint (**c**).

**Figure 2:**
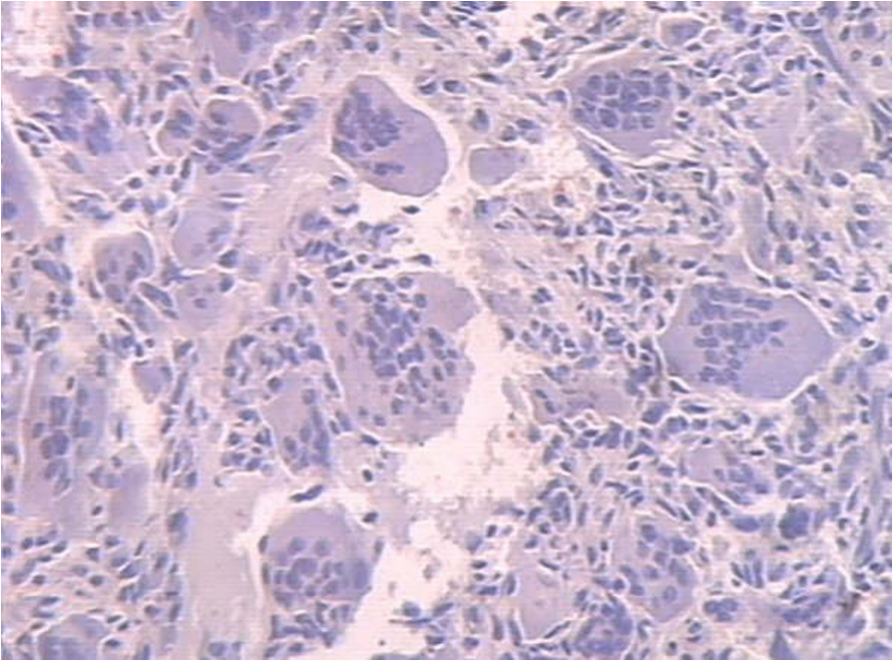
**Macroscopic features of the pathological specimen obtained by percutaneous puncture biopsy.** Typical appearance of giant cell tumor of bone with large osteoclast-like giant cells and uniform ovoid mononuclear cells (HE Hematoxylin & Eosin (HE) × 40).

**Figure 3:**
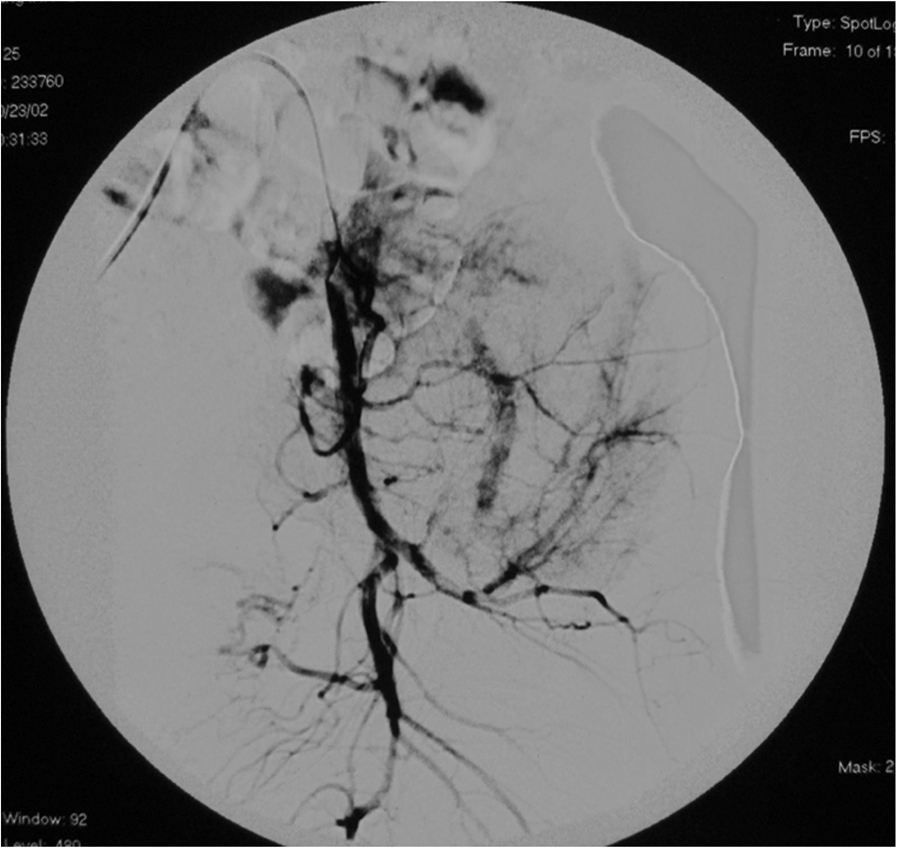
Arteriogram at the time of presentation shows markedly increased vascularity and uptake of contrast in the tumor.

**Figure 4:**
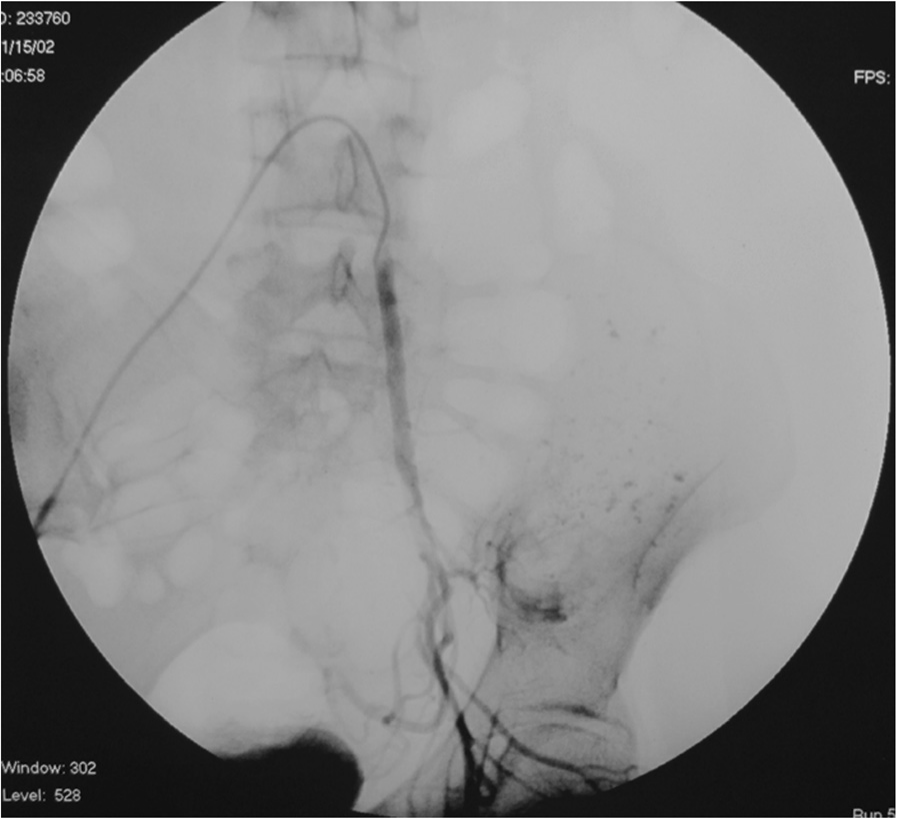
Arteriogram following the second embolization demonstrating the vascularity pattern of successful embolization.

A 31-year-old woman presented with severe pain in her left lower back and buttock, which severely restricted her gait. Radiography and computerized tomography (CT) revealed an eccentric geographic destructive osteolytic lesion involving the sacrum and the posterior superior iliac spine (Figure 1). A huge soft tissue mass had extended extra-osseously. After these imaging studies, the patient underwent a percutaneous puncture biopsy. The histological diagnosis was GCT of the bone (Figure 2). Intralesional embolization was performed using femoral access to selectively embolize the main arteries feeding the tumor. A catheter was advanced from the femoral artery into the internal iliac artery, and a selective angiogram was obtained to identify arteries of sufficient caliber to facilitate embolization. Injection of contrast medium showed a hypervascular, destructive tumor of the sacrum and ilium. Arteriography at the time of presentation showed markedly increased vascularity (Figure 3). These feeding branches were selectively embolized with 10 ml iodized oil and gelfoam particles. Embolization was performed again after 3 weeks and the total number of the embolizations was two (Figure 4).

**Figure 5:**
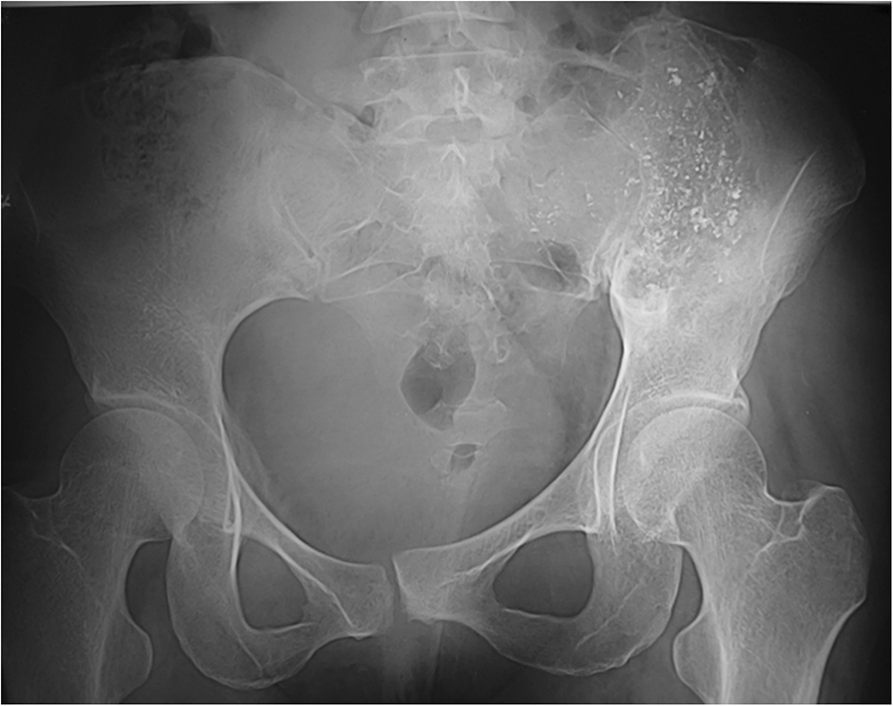
Radiography following the second embolization shows many iodized oil shadows in the osteolytic lesion.

**Figure 6:**
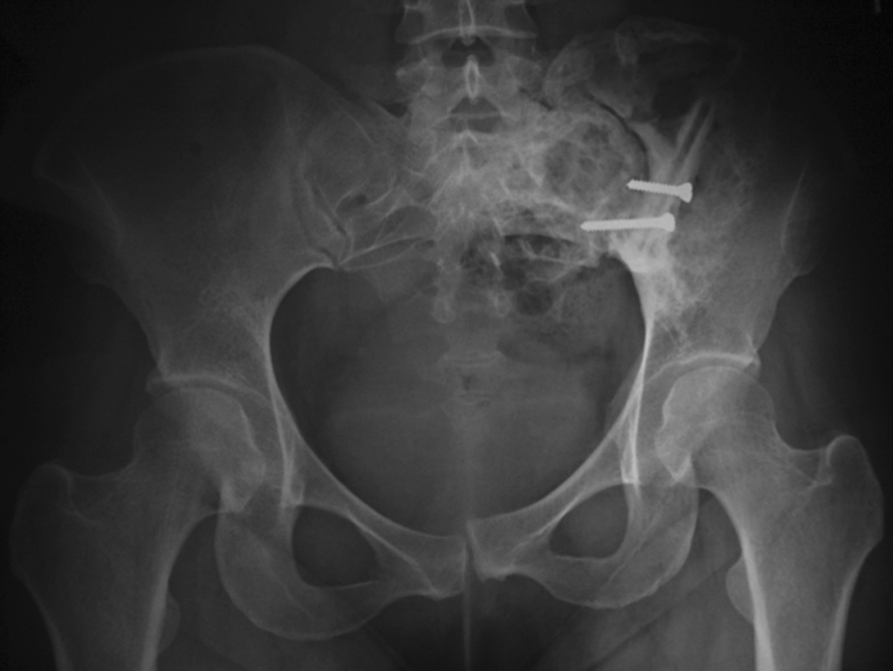
At the postoperative 9-year follow-up radiography shows slight displacement of the pubic symphysis and sacroiliac joint and good bone healing with no tumor recurrence.

After completion of the second embolization, the patient’s buttock pain improved significantly. Her gait became normal. Radiography demonstrated many iodized oil shadows in the osteolytic lesion involving the sacrum and ilium (Figure 5). Then she underwent tumor curettage and bone grafting with a combined anterior-posterior approach under general anesthesia. The first step was bilateral, internal, iliac artery ligation through the anterior approach, and the tumor was removed completely and bone grafting performed using the posterior approach. The blood loss in the patient was 10 units during the operation. At the final follow-up 9 years after completion of the management, radiography showed slight displacement of the pubic symphysis and sacroiliac joint, and good bone healing with no local recurrence (Figure 6). The patient returned to normal daily life activity.

## Discussion

GCT involving the sacrum and ilium presents a significant challenge and treatment methods, including surgical resection and radiation, and is associated with morbid complications and high recurrence rates [5,6]. Partial and/or total resection of the tumor, including the sacroiliac joint, can be an ideal therapeutic modality; however, it can result in significant morbidity, including neurological complication and infection, and mortality [7]. Intralesional curettage is the most common therapeutic option for GCT of the bone, especially for lesions of the extremities. However, the local recurrence rate after intralesional curettage is relatively high, and excessive intraoperative blood loss is often fatal [8]. More conservative surgical treatment has a high risk of local recurrence, from that of 23% to 33%, and even 100%, after curettage and cryotherapy [1].

Postoperative adjuvant therapy has a limited place in the treatment of GCT. Radiotherapy has been abandoned, since limited therapeutic benefit is combined with a risk of malignant change [1]. Embolization was initially used for inoperative primary or secondary bone tumors after failure of other forms of treatment [9]. Interest in arterial embolization of pelvic tumors followed its success as a palliative measure, particularly after resolution of pain and tumor shrinkage. As an alternative, minimally invasive and effective conservative treatment for GCT of the sacrum, selective arterial embolization, was introduced, and good long-term clinical results were demonstrated [4,7,8,10]. Hosalkar *et al*. [3] reported that seven of their nine cases had no disease progression at an average of 8.9 years of follow-up. Lin *et al*. [4] reported the clinical results for more than 10 years of follow-up of 18 patients treated with selective intra-arterial embolization. They demonstrated that the risks of local recurrence were 31% at 10 years, and 43% at 15 and 20 years, and concluded that the response after embolization was durable in about half of the patients. This technique has been shown to devascularize tumors, reduce their size, cause calcification of their margins and alleviate pain.

Previous reports demonstrated that a large population of patients responded favorably to intra-arterial embolization, with improvement in pain [1,3,4]. Rapid pain relief is one of the greatest advantages of intra-arterial embolization for GCT of the sacrum. With the treatment experience of five patients to examine the efficacy of serial arterial embolization, Lackman *et al*. [1] concluded that embolization was the primary therapy after histological confirmation of the diagnosis, which therefore obviated any exposure to chemotherapy or radiation. Our patient could not walk because of severe buttock pain at first presentation. This severe pain was probably due to destruction of the weight-bearing portion of the sacrum and ilium. After completion of the second embolization, her buttock pain improved significantly and her gait became normal. These rapid responses to the embolization led to the rapid pain relief in our patient.

These results prompted us to employ this therapeutic modality for our patient to decrease bleeding during the operation. After completion of preoperative selective arterial embolization (SAE), she underwent tumor curettage and bone grafting with a combined anterior-posterior approach. We had enough time to completely remove the tumor tissue and manage the tumor cavity to minimize blood loss. At the final 9-year postoperative follow-up, there was no local recurrence or metastasis, and the patient has returned to normal life. The good long-term outcome suggests that for this challenging disease, repeated preoperative SAE, curettage and bone grafting could be an effective and relatively less complicated technique, resulting in less blood loss.

## Conclusion

We think it is an optimal treatment for giant cell tumor involving the sacroiliac joint, with repeated selective arterial embolization and curettage, which has the advantage of less injury, less blood loss and fewer complications.

## Consent

Written informed consent was obtained from the patient for publication of this Case report and any accompanying images. A copy of the written consent is available for review by the Editor of this journal.

## Abbreviations

CT: Computerized tomography; GCT: Giant cell tumor; RSAE: Repeated selective arterial embolization; SAE: Selective arterial emolization

## Competing interests

The authors declare that they have no competing interests.

## Authors’ contributions

YXC led the coordination of this case report. FZH has assisted with the embolization operation. XM and XSF have made great effort in the follow-up of this patient. YXC wrote this manuscript. All authors participated in the modification of drafts and read and approved the final manuscript.
